# Managing hand and reconstructive microsurgery service during COVID-19 pandemic: Singapore experience

**DOI:** 10.1136/postgradmedj-2020-137735

**Published:** 2020-06-10

**Authors:** Usama Farghaly Omar, Tong Pei Yein, Vaikunthan Rajaratnam

**Affiliations:** Department of Orthopaedic Surgery, Khoo Teck Puat Hospital, Singapore; Department of Orthopaedic Surgery, Khoo Teck Puat Hospital, Singapore; Department of Orthopaedic Surgery, Khoo Teck Puat Hospital, Singapore

**Keywords:** infectious diseases, hand & wrist, respiratory infections, infection control, limb reconstruction, plastic & reconstructive surgery

## Abstract

**Introduction:**

Managing healthcare service during pandemics and outbreaks is a challenging process. The aim is to keep patient safety as the priority, besides, continuing to provide essential healthcare services.

**Methods:**

Situational audit was performed for the services rendered before and during COVID-19 pandemic and the elevation of the disease alert status, and a retrospective analysis of the attendance and procedures performed in the service.

**Results:**

We present a methodology for performing a situational audit and generating service modification for hand and reconstructive microsurgery unit in a pandemic. There was no significant difference between the number of patients seen at outpatient clinics. However, there was a reduction in the numbers of total surgeries performed, with a 40% drop in the number of elective surgeries performed. There was also a reduction of cases seen in the emergency department hand clinic.

**Discussion:**

COVID-19 pandemic is currently affecting not only the health service but also, other vital services all over the world. The pandemic puts significant challenges to acute surgical services in a hospital system involved in the management of the pandemic. Surgeons need to take proactive and a systematic approach in managing the available resources while maintaining essential surgical services. This paper provides the tools and methodology for doctors to plan their services in a pandemic situation.

**Conclusions:**

It is possible to maintain essential surgical services in a pandemic situation through rapid situational audits and generating localised strategies while considering the constraints imposed during the pandemics while maintaining patient and staff safety.

## Introduction

Since the first reported COVID-19 on 31 December 2019, by China,^1^ the disease has spread both locally and outside China, first in Asia and then globally. WHO on the 12 March 2020 declared COVID-19 as a pandemic noted 125 048 confirmed cases globally with 6729 new cases in the preceding 24 hours, 4613 deaths (321 new), and in China 80 981 confirmed cases (26 new) and 3171 deaths (11 new).This is the first pandemic to be caused by coronavirus. WHO risk assessment for COVID 19 is very high globally.^2^ Latest update by the Ministry of Health (MOH) on 8 May 2020 showed the total number of cases 21 707 (768 new), deaths 20 (no new deaths). Despite the rapid increase in the total number of reported cases, only 1245 (22 in ICU - Intensive Care Units) are hospitalised and total discharged are 2040 while the remaining 18 402 are isolated in community facilities (tested positive but showing no symptoms).^3^

As China’s cases continue to stabilise, globally the number of new cases continued to grow. China’s control measures included the lockdown in Hubei province, which restricted household transmission leading to the burn-out of the virus. However, these stringent strategies may not be reproducible in other countries with different governance systems.^4  5^ Also, on 9 March 2020, Italy imposed national quarantine over the whole country and restricted movement of the population through the country except for essential travel for work and health issues. Rome airports terminals are closed.^6  7^

Historical viral outbreaks of significance include SARS-CoV (severe acute respiratory syndrome coronavirus) epidemic affected 26 countries in 2003. Singapore was one of the countries severely affected by the outbreak.^8^ In 2009, the influenza A (H1N1-2009) outbreak in Singapore was met with a disease surveillance system and influenza pandemic preparedness plan, following the SARS outbreak. Known as the Disease Outbreak Response System Condition (DORSCON), it is a five-colour alert system that progresses from green to yellow, orange, red and black. Table 1 summarises the different DORSCON levels.^9^ The key learning points from that H1N1 influenza pandemic in Singapore were as follows^10^:

**Table 1:** Different DORSCON levels

	Green	Yellow	Orange	Red
Nature of the disease	The disease is mild. Or The disease is severe but does not spread easily from one person to another.	The disease is severe and easily spread from one person to another but occurring outside Singapore. Or The disease is spreading in Singapore but: Typically, mild: only slightly more severe than seasonal influenza, could be severe in vulnerable groups. Being contained.	The disease is severe and easily spread from one person to another, but the disease has not widely spread in Singapore and being contained.	The disease is severe and spread widely.
Impact on daily life	Minimal disruption: border screening and travel advice.	Minimal disruption: additional measures at borders and/or healthcare settings expected, higher work and school absenteeism likely.	Moderate disruption: temperature screening, quarantine and visitors’ restrictions at hospitals.	Major disruption: School closures, works from home orders, significant numbers of deaths.
Advice to public	Be socially responsible: if you are sick stay at home. Maintain good personal hygiene. Look out for health advisories.	Be socially responsible: if you are sick stay at home. Maintain good personal hygiene. Look out for health advisories.	Be socially responsible: if you are sick stay at home. Maintain good personal hygiene. Look out for health advisories. Comply with control measures.	Be socially responsible: if you are sick stay at home. Maintain good personal hygiene. Look out for health advisories. Comply with control measures. Practice social distancing: avoid crowded areas.

DORSCON, Disease Outbreak Response System Condition.

1. Be prepared but retain flexibility in implementing control measures.
2. Surveillance, good scientific information and operational research can increase a system’s ability to manage risk during a public health crisis.
3. Integrated systems-level responses are essential for a coherent public health response.
4. Effective handling of manpower surges requires creative strategies.
5. Communication must be strategic, timely, concise and clear.

On 23 January 2020, the first case of COVID-19 was announced in Singapore. As the infections spread, on 7 February 2020, MOH escalated the DORSCON to orange alert level with 33 cases confirmed in Singapore. In March 2020, 80% of the cases were imported from returning Singaporeans, however, in April 2020, there was an increasing proportion of unlinked cases, indicating underlying community transmission.^3^ On 7 April 2020, the government imposed circuit breaker and further stricter measures on 21 April 2020 with an extension to 1 June 2020.^11^ These measures included the shutdown of all schools, universities and non-essential services. All front-lines staff went into business continuity plan, where manpower is split into teams, with each team working for a determined period on-site and then remain on standby at home for the same period while the other team works. All non-essential industries implemented work from home practices.^12  13^ Contact tracing and cluster identification remain one of the main mitigation measures in Singapore.

This COVID-19 pandemic appeared to be more contagious than previous SARS and MERS (Middle East respiratory syndrome) and affecting multiple countries and has a significant impact on healthcare systems. It is expected to last for at least 3 months. There needs to be more collaboration between various agencies including the private and public sector to overcome the Pandemic.^14^ The impact on the healthcare systems varies from increased demand in care of patients infected, the potential for the workforce to be affected from exposure to the infection both in themselves and their families and from social distancing measures. This will compound the efficiency of the delivery of healthcare services. One of the framework in responding to pandemics is the use of pandemic exercise scenario.^15^ One of the suggested strategies includes the use of telemedicine and reducing unnecessary contact with non-urgent cases within the healthcare facility.^16^ Singapore’s response to COVID-19 included the following^17^:

- Surveillance systems were readjusted to identify potential cases.
- Selectively control travellers entering Singapore and travelling to infected locations.
- Intragovernmental coordination.
- Developed plans to sustain routine healthcare services.
- Critical care treatment and medicines made available for patients with COVID-19.
- Training and adherence to infection prevention and control measures in hospitals.
- Adapted financing measures so that all direct costs for treating patients are borne by the governments. In Singapore, the government pays the cost of hospitalisation, irrespective of whether the patient is from Singapore or abroad.
- Management of information systems is comprehensive accurate and transparent risk communication.

Singapore health authorities provide daily information on various platforms including mainstream media, the MOH has Telegram and WhatsApp for public and groups set up with doctors in the public and private sectors where more detailed clinical and logistics information is shared, and authorities use websites to debunk circulating misinformation.^18^ Singapore implemented a mobile contact tracing app called TraceTogether. The OneService app developed for reporting of municipal issues was repurposed to report any noncompliance with safe distancing.^19–22^ In addition, all national and international scientific events were cancelled as well as overseas travel for all healthcare staff in the public sector. Temperature and travel screening and reporting were conducted at public places and restaurants. Healthcare staff of public hospitals were required to declare their travel history and record their body temperature twice daily and report it to the MOH. It was declared by MOH that for the public, wearing a surgical mask is enough and only when feeling sick or having upper respiratory tract symptoms; this was later extended to all public when outside their homes during circuit breaker period. Singaporean government dispatched four surgical masks for each household to be used only if one feels sick or in case seeing a doctor. MOH also stated that crew and front-line medical staff specially those who are doing screening for patients or dealing with infectious cases should wear personal protective equipment (PPE) including goggles, N95 mask, gowns, gloves and caps.^12  23^

### Methodology

This is a narrative observational account of the various measures undertaken at a public hospital and its impact in the delivery of an acute and elective surgical service. This study aims to describe and document the process of auditing the services of a hand and reconstructive microsurgery (HRM) and design and develop strategies to ensure the continuity of HRM services required by the community. This study was conducted at Khoo Teck Puat Hospital, a 761-bed hospital that serves the northern part of Singapore, where more than half a million people live. HRM services are provided within the orthopaedic surgery department. This comprehensive service includes the provision of acute and elective services for conditions pertaining to the upper limb and microsurgical and soft tissue reconstruction. Before the pandemic of COVID-19, the service includes the provision of outpatient services 4 days in a week, a 5-day emergency hand service clinic in the emergency department (ED) from 17:00–22:00 hours (Hot Hand Clinic), 3 days operating of emergency (P4 system) and elective services in a week. This was further augmented with weekly audits and continuing professional development meetings and monthly research meetings.

The situational audit was performed of the services rendered prior to, during COVID-19 pandemic (DORSCON orange level) and COVID-19 pandemic (Circuit breaker), and a retrospective analysis of the attendance and procedures performed in the service.

## Results

The average number of patients seen in the clinic before the pandemic was 900/month. The average number of operated patients was 150/month and average patients seen at ED with acute problems was 75/month. Weekly audit and teaching activity were held to discuss preoperative and postoperative cases in addition to literature updates in hand and reconstructive surgery.

With the escalation by the MOH to DORSCON Orange status, there was a need for an urgent restructuring of services that could be delivered safely and in keeping with the requirements of the elevated level of pandemic alert.

### Manpower management

There was cancellation of all types of staff leave except for sick leave and emergency leave. HRM specialists, as well as orthopaedic and sports medicine specialists, were split into teams. Each team composed of two consultants as preparation for deployment to pneumonia wards.

### Education

All educational events of the HRM were cancelled. An international conference on poststroke spasticity organised by HRM unit in Okinawa in December 2020 required the use of teleconference facilities for committee meetings and the decision was made to postpone this event to 2021.

### Specialist outpatient clinics

To reduce the number of patient visits to the hospital, all outpatient appointments were triaged by HRM medical staff and only urgent cases were given appointments and the rest was postponed to post circuit breaker. All patients were offered teleconsultation for reassurance and repeat medication. Social distancing was addressed between patients in the waiting area. All patients were instructed to wear a mask for hospital attendance. Waiting areas were restricted for patients. Consultation rooms had minor changes in the form of one room was assigned for teleconsultation and one room was assigned for consulting COVID-19 suspected cases using full PPE.

With the emergence of the second wave, tighter restrictions have been announced as a part of the circuit breaker. Table 2 addresses the differences between HRM services, activities, and manpower before and during COVID-19 pandemic. While table 3 shows the effect of COVID-19 over HRM unit workflow.

**Table 2:** Changes in service types done by HRM before and after DORSCON orange

Service type	Prepandemic	Changes during COVID-19 pandemic (DORSCON orange level)	Changes during COVID-19 pandemic (circuit bBreaker)
**Clinics**	Monday, Tuesday, Wednesday and Thursday. Accepting both elective and emergency referrals Operated by HRM staff and Hand occupational therapists	Same working days as prepandemic but: Reduced to postsurgery, postinjury cases and urgent referrals only. Triage of elective referrals to be rescheduled after lowering of the national alert level.	Monday to Friday clinic Cancellation of specialist hand clinic, all orthopaedic subspecialties are managed through the general orthopaedic clinic.
**Operative service**	Dedicated HRM operative theatres as follows: Monday: one operative theatre Wednesday: one operative theatre Friday: two operative theatres Both elective and emergency surgeries were performed.	Same Operative days with the following changes: Only emergency cases Cancellation of all elective surgeries Tele counselling of all cancellations by HRM staff Registry of cancelled cases maintained. Removal of dedicated HRM operative theatres. Prioritisation of emergency cases done by the theatre management team.	Same policy as DORSCON orange level but with following add-ons: General emergency hand cases were managed by general orthopaedic staff. Complex cases were operated by HRM staff. Listing of patients monitored closely by operating theatre management team. Strict penalties imposed by the Ministry of Health for failure to adhere to the policy
**Emergency service**	Hot hand clinics in ED Monday to Friday. 8:30 to before 17:00 cases screened by ED doctors before referring to HRM staff. 5:00–10:00 all cases are referred directly from triage nurses to HRM staff on site. Minor procedures (simple debridement and stitches) performed in ED. Complex cases managed by P4 system (a day care semiemergency) delivered Monday, Wednesday, Friday.	Hot clinics in ED Monday to Friday: 8:30 till 22:00 cases screened first by ED doctors before referring to HRM staff or teleconsultation done by ED doctors using TigerConnect* software. Cancellation of minor procedures in ED. All simple and complex cases are managed by P4/system and operated on Monday to Friday.	Hot hand clinic cancelled. All hand cases are managed as a part of the general orthopaedic emergency call. All simple and complex cases are managed and operated 24/7.
**Audit/CME**	Thursday, multidisciplinary team, Lunch time meeting	Use of TigerConnect software, face-to-face ward rounds and during performing surgeries.	Same as DORSCON orange level policy
**Conference attendance**	Attendance (FESSH, APFSSH, Local and regional meetings)	Cancellation, use video conferencing	Same as DORSCON orange level policy
**Undergraduate teaching**	Regular tutorials NUS and NTU year 3 and final years	Cancelled (use available online resources)	Same as DORSCON orange level policy
**Postgraduate teaching**	Friday PM face to face teaching Daily residence work-based teaching	Friday PM teleconferencing teaching Daily work-based teaching in small groups face-to-face teaching with social distancing	Friday PM teleconferencing only. Cancellation of all face-to-face meetings.
**Multicentres meeting and research projects**	Monthly face to face meetings. Cross-site collaborative research. Weekly research clinic to review patient included in projects.	Cancellation of all face-to-face meetings Digital messaging and teleconferencing. In order to comply with physical distancing measures, all research projects involving our off-site biomechanical laboratory partners and prospective studies have been consolidated. Conduct retrospective studies or literature and systematic reviews. Cross-site physical collaborative research cancelled. Weekly research clinic cancelled.	Same as DORSCON orange-level policy
**External meetings**	Organ transplant ethics National medical ethics Academic/exams	Organ transplant committee teleconference. National medical ethics meeting Cancelled Held by teleconferencing.	Same as DORSCON orange-level policy
**Manpower**	HRM team consists of 2 consultants, 2 resident physicians, 1 senior resident, 1 junior resident or medical officer, 1 house officer and one clinical fellow. Manpower was planned based on a monthly roster.	The team were segregated into two groups, alternating between operative theatre duties and inpatients and clinics duties weekly Manpower was planned based on a weekly roster.	Cessation of specialty HRM team Single general orthopaedic service Implementation of two BCP teams: alternating between active duties on-site and standby 2 weekly. Some staff were deployed for COVID 19 screening or other essential services in ED and wards.

*TigerConnect: it is an online text messaging and media application which is accessed only using the institutional email account. It is used as an official way of handling patients details specially those containing personal data.

APFSSH, Asian Pacific Federation of Societies for Surgeries of The Hand; BCP, Business continuity plan; DORSCON, Disease Outbreak Response System Condition; ED, emergency department; FESSH, Federation of European Societies for Surgery of the Hand; HRM, hand and reconstructive microsurgery; NTU, Nanyang Technological University; NUS, National University of Singapore.

**Table 3:** Changes affecting HRM unit workflow in KTPH before and after COVID-19

	November, December 2019 (before COVID-19)	January, February 2020 (during COVID-19)	P value
Outpatient clinic cases	1303	1323	0.34
Admissions	23	25	0.33
Total no of operated cases	196	144	0.0014*
Operated emergency cases	94	77	0.085
Operated elective cases	102	67	0.012*
Patients were seen at Hot Hand Clinic	93	73	0.0061*
Procedures were done at ED	32	0	–

*P<0.05 is significant.

ED, emergency department; HRM, hand and reconstructive microsurgery; KTPH, Khoo Teck Puat Hospital.

## Discussion

Our operative case mix was significantly affected, with a shift from 61% elective and 39% emergency surgeries^24^ in the prepandemic period, to 46.5% elective and 53.5% for emergency surgeries during the pandemic period. There was no significant change in outpatient flow despite the postponement of all elective consultation. This was due to the increased flow of day care semiemergency cases that required postoperative reviews in the outpatient. The number of cases seen in the Hot Hand Clinic showed some reduction due to suspension of the service and selective referral to the HRM by the ED. As per elective surgery cancellation policy, 22 patients were cancelled.

Continued delivery of urgent and emergency hand surgery service led to an increase in numbers of postoperative follow-up cases in the clinic. Also, cases managed by ED doctors were referred for follow-up in the hand clinic. Thus, the drop in elective cases did not affect the total number significantly. There was a decrease in the number of procedures done in ED, following the cancellation of the routine emergency HRM service at the ED from 8:30–22:00 hours, to minimise exposure of HRM staff. However, ED doctors were given teleconsultation with HRM service through TigerConnect supported by clinical data including clinical images.

HRM unit maintains an online educational resource on HRM even before the COVID-19 (www.handsurgeryedu.com and a YouTube Channel Hand Surgery International). As all department educational events and case presentations were cancelled, these online platforms were used to provide for continuing education.

All cancelled elective cases were tracked and recorded electronically for priority relisting once the elective surgery is commenced. All postponed non-urgent outpatient clinic appointments will be offered teleconsultation and another physical appointment once the outpatient flow returns to pre-pandemic status.

In the event of a national pandemic with international implications, the role of the hospital is crucial and requires specific strategies to cope with the emergency while at the same time maintaining a level of service to meet the needs of the community.

Hand injuries and emergencies continue to be one of the major burdens at the ED worldwide. Challenges during a pandemic are to balance the allocation of resources between routine and emergency needs and should be ethically sound and socially just.

As our hospital is part of the national public health system, collaboration and cooperation between the MOH, the national primary care system and the hospital is already well developed. It is important to perform a situational audit of all the current services in the specialty and align these with that of the needs and requirements of the pandemic in consultation with the national pandemic command centre. Once the situational audit has been performed, strategies can be generated to modify the current services to meet the demands and needs of the community taking into consideration the needs of the pandemic. The essential routine services must be maintained, and the deferment of elective services should be balanced carefully with a phased approach to consider how it will be restarted once the pandemic is over. The strategies must include the identifications of the required skill sets and numbers and seniority of the personnel to ensure the continuity of the essential service.

To reduce the risk of pandemic transmission in the hospital, the patient workflow must be reorganised to avoid contact between patients requiring routine essential care and those affected by the pandemic.

## Conclusion

During any pandemic, the emphasis on most healthcare processes has been in the containment and treatment of the pandemic. However, in most multidiscipline hospitals, essential services that are not related to the pandemic need to be continued to be provided at a level to meet the needs and demands of the community

In this paper, we have looked at the methodology on analysing the services provided in HRM in a multidiscipline hospital during the pandemic of COVID-19. This useful methodology of providing a situational audit and generating strategies to continue essential service while managing a pandemic. It is important while providing a service to make sure that the uninfected patients and staff are kept safe while delivering essential services. Ensuring seamless communication between the team members remotely and face to face allows for an integrated approach towards delivery of care. The use of technology and innovative ways of delivering care with fewer resources and capitalising on unused resources during pandemics are the principles of modifying services to ensure continuity of essential services.
